# Inflammation Is Associated with Worse Outcome in the Whole Cohort but with Better Outcome in Triple-Negative Subtype of Breast Cancer Patients

**DOI:** 10.1155/2020/5618786

**Published:** 2020-12-08

**Authors:** Masanori Oshi, Stephanie Newman, Yoshihisa Tokumaru, Li Yan, Ryusei Matsuyama, Itaru Endo, Kazuaki Takabe

**Affiliations:** ^1^Department of Surgical Oncology, Roswell Park Comprehensive Cancer Center, Buffalo, New York 14263, USA; ^2^Department of Gastroenterological Surgery, Yokohama City University Graduate School of Medicine, Yokohama 236-0004, Japan; ^3^Department of Surgery, Jacobs School of Medicine and Biomedical Sciences, State University of New York, Buffalo, New York 14263, USA; ^4^Department of Surgical Oncology, Graduate School of Medicine, Gifu University, 1-1 Yanagido, Gifu 501-1194, Japan; ^5^Department of Biostatistics & Bioinformatics, Roswell Park Comprehensive Cancer Center, Buffalo, New York 14263, USA; ^6^Department of Gastrointestinal Tract Surgery, Fukushima Medical University School of Medicine, Fukushima 960-1295, Japan; ^7^Department of Surgery, Niigata University Graduate School of Medical and Dental Sciences, Niigata 951-8510, Japan; ^8^Department of Breast Surgery and Oncology, Tokyo Medical University, Tokyo 160-8402, Japan

## Abstract

Inflammation has been linked with cancer, but whether it is part of the problem or part of the solution remains to be a matter of debate in breast cancer. Our group and others have demonstrated that inflammation aggravates cancer progression; however, some claim that inflammation may support immune cell infiltration and suppress cancer. We defined the gene set variation analysis of the Molecular Signatures Database Hallmark inflammatory response gene set as the inflammatory pathway score and analyzed 3632 tumors in total from 4 breast cancer cohorts (METABRIC, TCGA, GSE25066, and GSE21094). In the whole breast cancer cohort, high-score tumors were associated with aggressive clinical characteristics, such as worse disease specific survival, higher Nottingham histological grade, and younger age. Inflammatory score was significantly higher in triple-negative (TNBC) as well as basal and normal subtypes compared with the other subtypes, which suggest that the detrimental effect of high level of inflammation may be because it includes a more aggressive subtype. On the contrary, high score within TNBC was significantly associated with better survival. TNBC with high score enriched not only IFN-*α*, IFN-*γ* response, IL-2/STAT5 signaling, Allograft rejection, Complement, p53 pathway, Reactive Oxygen, and Apoptosis but also TNF-*α* signaling, IL6-JAK-STAT signaling, TGF-*β* signaling, Coagulation, Angiogenesis, EMT, *KRAS* signaling, and PI3K-AKT-MTOR signaling gene sets. High score was associated with mainly favorable anticancerous immune cell infiltration as well as Leukocyte fraction, TIL regional fraction, Lymphocyte infiltration, IFN-*γ* response, TGF-*β* response, and cytolytic activity scores. Although the inflammatory pathway score was not associated with neoadjuvant treatment response, it associated with expressions of immune checkpoint molecules. In conclusion, inflammation was associated with worse outcome in the whole breast cancer cohort, but with better outcome in TNBC, which was associated with favorable anticancerous immune response and immune cell infiltrations.

## 1. Introduction

The link between inflammation and cancer was first suggested in 1863 [1], but yet, whether inflammation aggravates or conciliates cancer remains the matter of debate to date. William Coley, a surgeon, observed regression of cancer in patients infected with erysipelas and injected bacterial toxin to treat cancer in 1891, known as “Coley toxin” [2]. On the other hand, chronic inflammation is known to increase the risk of cancer development, such as colon cancer in inflammatory bowel diseases [3]. Recently, we have published that sphingosine-1-phosphate (S1P), produced by sphingosine kinase 1 (*SphK1*) in breast cancer, signals through S1P receptor 1 (*S1PR1*) and activates the amplification loop of interleukin-6 (*IL-6*) and tumor necrosis factor *α* (*TNFα*) in obesity-related inflammation, which promotes lung metastasis and worsens survival as well as increases resistance to therapy [4–6].

The exact nature of the inflammation has been implicated to have both anticancerous and procancerous effects [7]. High density of tumor infiltrating T cells (TILs) is a strong predictor for patient's better survival [8, 9], whereas the abundance of regulatory T cells is associated with poor prognosis [10]. M1 macrophages secrete interferon-gamma (IFN-*γ*), which has an anticancerous effect, whereas tumor-infiltrating M2 macrophages are procancerous and are associated with tumor growth and metastasis [11–13]. Infiltration of inflammatory cells such as T helper type 1 (Th1) cells were reported to be a predictor of better survival in breast cancer patients [14–16]. Proinflammatory cytokines have a similar ambiguous role; high-dose interleukin-2 (IL-2) has been used to treat melanoma, whereas *TNFα* is associated with aggressive breast cancer biology [17], and its inhibitor, etanercept, is currently tested in the phase II clinical trial for metastatic breast cancer [18]. These inflammatory mediators directly affect both cancer and stromal cells and contribute to several hallmarks of cancer, such as promotion of the epithelial-to-mesenchymal transition (EMT) and metastasis of cancer cells [19, 20]. Breast cancer is clinically divided into subtypes based on molecular expression of biomarkers: estrogen receptor (ER), progesterone receptor (PR), and human epidermal growth factor receptor 2 (HER2). ER-positive breast cancers constitute the most common subtype representing approximately 70% of breast cancer, which have relatively better survival compared to the other subtypes. On the other hand, triple-negative breast cancer (TNBC), the most aggressive subtype, is known to accompany inflammation in the stroma with infiltration of immune cells more frequently than the other subtypes [21]; however, whether the inflammation is part of the problem or part of the solution is to be determined in this setting as well.

One of the challenges in studying the role of inflammation in cancer particularly in clinical setting is the difficulty in quantification of the inflammation. Currently, inflammation is commonly evaluated by pathological analyses of the number of infiltrating mononuclear cells by hematoxylin and eosin stain (H&E) or immunohistochemistry (IHC) [22, 23]. Given the wide variety of cells and cytokines involved in inflammation, whether counting several types of immune cells allows thorough understanding of the entire picture of inflammation is highly questionable in addition to the concern over subjective nature of the pathological quantification.

Lately, we have been reporting the clinical relevance of signaling pathways in cancer using the gene set variation analysis (GSVA) of the transcriptome of bulk tumors. GSVA analyzes hundreds of genes related to a pathway as a score that estimate the degree of the activation of the pathway. We have reported the role of *KRAS* signaling in TNBC [24] and the role of G2M cell cycle pathway in estrogen receptor- (ER) positive breast cancer [25]. GSVA-based pathway score approach takes coordination of genes into account, reduce model complexity, and increase the explanatory power of prediction models.

Here, we hypothesized that inflammation, evaluated by inflammatory pathway score based on GSVA of 200 inflammatory pathway-related genes of tumor transcriptome, associates with aggressive cancer biology and worse survival.

## 2. Materials and Methods

### 2.1. Breast Cancer Cohorts and Their Data

Tumoral genomic profiling and clinical information of TCGA-BRCA (*n* = 1065) [26] and METABRIC (*n* = 1903) [27] cohorts were provided the cBio Cancer Genomic Portal [28]. Furthermore, we used normalized tumoral genomic and clinical data provided by Gene Expression Omnibus (GEO) repository of the US National Institutes of Health. For genes with multiple probes, the average value was used. Gene expression data were transformed for log_2_ in all analysis. We used published data of Shi et al. (GSE20194; *n* = 248) [29] and Symmans et al. (GSE25066; *n* = 467) [30] to investigate the association of the inflammatory scores with treatment response for neoadjuvant chemotherapy.

### 2.2. Gene Set Expression Analyses

We utilized the gene set variation analysis (GSVA) method [31] to measure the inflammatory pathway score as the GSVA score of the inflammatory response gene set of the MSigDB Hallmark collection [32] using the GSVA Bioconductor package (version 3.10). Within-cohort median values were used to assign low and high inflammatory pathway score. The statistical significance of false discovery rate (FDR) in the GSEA analysis was less than 0.25 recommended by GSEA software (Lava version 4.0), as we previously reported [33–47].

### 2.3. Other

R software (version 3.6.2, R Project for Statistical Computing) and Excel (version 16 for Windows; Microsoft, Redmond, WA) were used to perform mRNA data analysis and make figures. Patients with more than the median value of the inflammatory score were considered the high inflammatory score group, and the remaining patients were stratified into the low inflammatory score group. Patients were grouped by AJCC stage (I-IV), tumor size (T1-4), and lymph node metastasis (N0-3) for comparison analysis. We used log-rank test and Kaplan-Meier method to examine the associations with disease free survival (DFS), disease specific survival (DSS), overall survival (OS), and the inflammatory pathway score. The log-rank test was used to compare the differences in these survival analyses between patients from the two groups. The xCell algorithm was used for tumor composition analysis of infiltrated immune cells based on tumor mRNA data [48]. The statistical significance was taken as *p* < 0.05 of differential results. Analysis of variance (ANOVA) or Fisher's exact tests were used to provide statistical comparisons between groups. In the data diagram, Tukey-type boxplots shows median and interquartile level values.

## 3. Results

### 3.1. High Inflammatory Pathway Score Is Associated with Worse Clinical Features in the Whole Breast Cancer Cohort

**Figure 1:**
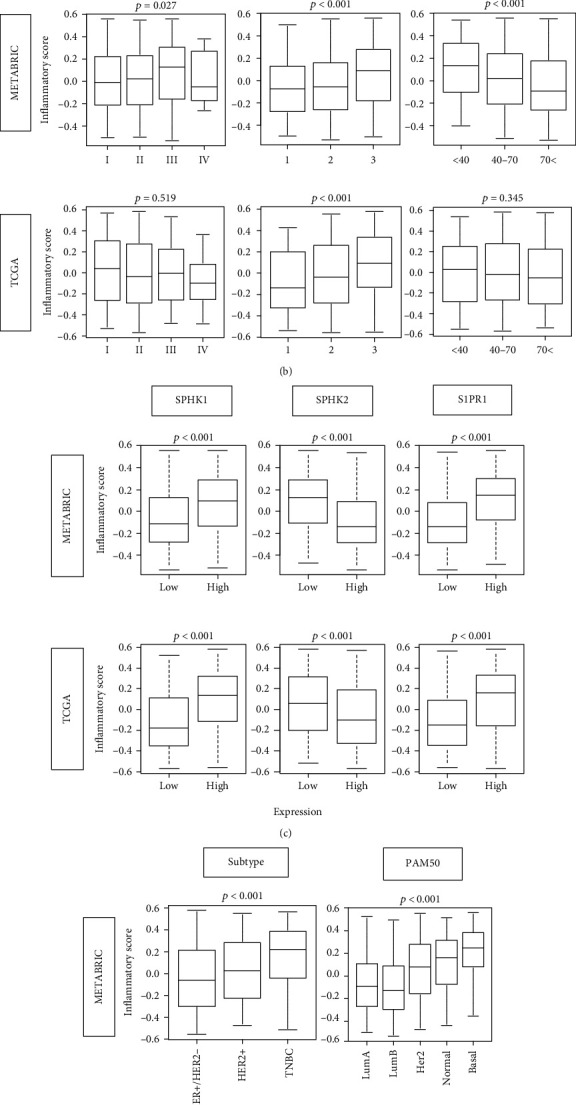
Association between inflammatory pathway score and the clinical factors in breast cancer. (a) Disease-specific (DSS), disease-free (DFS), and overall survival (OS) of inflammatory score high (red line) and low (blue line) in whole METABRIC breast cancer cohort. Log-rank test was used to compare the groups with Kaplan-Meier survival curves. (b) Boxplots of the inflammatory scores by AJCC cancer stage, Nottingham pathological grade, and age at diagnosis (<40 yo, 40-70 yo, and <70 yo) in both METABRIC and TCGA cohorts. (c) Gene expression levels of SphK1, SphK2, and S1P receptor 1 by high vs. low inflammatory score. (d) Boxplots of the inflammatory scores by subtype and PAM50 classifications. Tukey-type boxplots show median and interquartile level values, and the ANOVA test is used to calculate *p* values.

We defined the GSVA score of the Molecular Signatures Database (MSigDB) Hallmark inflammatory response gene set as the inflammatory pathway score (see Supplemental Table 1 for the genes included in the gene set). The cohorts were divided into high- and low-score groups by median value. We hypothesized that the high degree of inflammation is associated with clinical aggressiveness of breast cancer and worse survival. To test this hypothesis, we analyzed the disease-specific (DSS), disease-free (DFS), and overall survival (OS) of high vs. low inflammatory pathway score groups (Figure1(a)). High-score group was associated with worse DSS (*p* = 0.002), but not with DFS (*p* = 0.232) or OS (*p* = 0.257) in the Molecular Taxonomy of Breast Cancer International Consortium (METABRIC) cohort. We next examined whether there was an association between the inflammatory score and clinical features of aggressive breast cancer. The inflammatory score was significantly higher in advanced American Joint Committee on Cancer (AJCC) cancer staging in METABRIC, but not in The Cancer Genome Atlas (TCGA) cohort (Figure 1(b); *p* = 0.027 and *p* = 0.519, respectively). The score was also not associated with tumor size (pathological T category) nor lymph node metastasis (pathological N category) in the TCGA cohort (Figure S1A). On the other hand, inflammatory scores were uniformly high in higher Nottingham pathological grade in both cohorts (both *p* < 0.001). Further, inflammatory scores were significantly higher in younger patients, aged less than 40 years old in the METABRIC cohort, which is known to have aggressive cancer biology, but not in the TCGA cohort (Figure 1(b); METABRIC *p* < 0.001, TCGA *p* = 0.345). Since the amount of adipose tissue may have a strong impact on the type of immune response associated to the tumor [49], we investigated the association of the score with body mass index (BMI) using available data of the TCGA cohort (*n* = 81). There was no significant difference in inflammation score by BMI (Figure S1B; *p* = 0.836).

Given that a lipid mediator S1P links cancer and inflammation, it was of interest to study the association between the expression of S1P signaling-related genes and inflammatory score in human breast cancer cohorts. High expression of SphK1, which exports S1P out of the cell, was associated with higher inflammatory score with the striking consistency in the two cohorts (Figure 1(c); both *p* < 0.001). In agreement, sphingosine kinase 2 (*SphK2*) low-expression tumors, which often cause compensatory increase in SphK1 expression, were also associated with higher inflammatory score in both cohorts (both *p* < 0.001). Furthermore, inflammatory score was significantly elevated in tumors that express high levels of S1P receptor 1 (*S1PR1*), the S1P-specific receptor known to participate in cell growth, migration, and angiogenesis (both *p* < 0.001).

Given the significant relationship of high inflammatory score and aggressive clinical features, it was of interest to study whether subtype of breast cancer associates with score. Indeed, we found that inflammatory score was significantly higher in the TNBC, the known most aggressive subtype, compared with other subtypes defined by IHC, which was consistent in both METABRIC and TCGA cohorts (Figure 1(d); both *p* < 0.001). The inflammatory score was also significantly higher in the PAM50 classification of basal and normal subtypes, which are known to be clinically aggressive, once again in both METABRIC and TCGA cohorts (Figure 1(d); both *p* < 0.001). These results suggest that inflammation is associated with breast cancer aggressiveness and worse DSS most likely because aggressive TNBC subtype is included in inflammation high group.

### 3.2. High Inflammatory Score in TNBC Patients Is Significantly Associated with Better Survival

**Figure 2:**
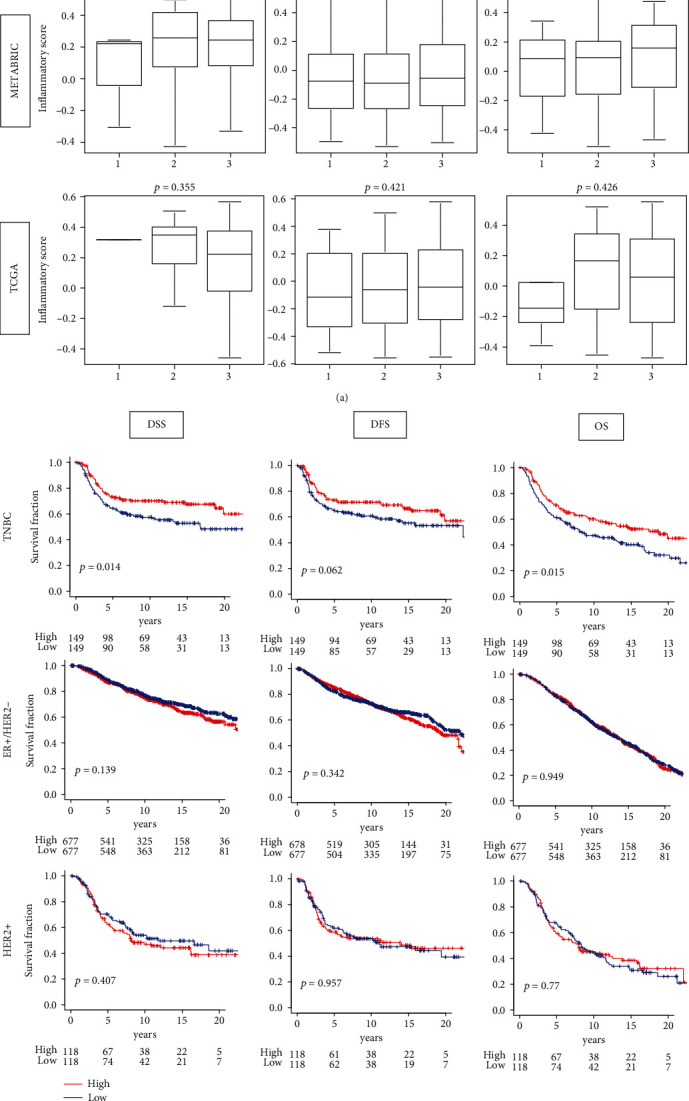
Association between the inflammatory pathway score and tumor aggressiveness by the subtypes. (a) Boxplots of the inflammatory scores by Nottingham pathological grade in triple-negative breast cancer (TNBC), estrogen receptor-positive/human epidermal growth factor receptor 2-negative (ER+/HER2-), and HER2-positive subtypes of the METABRIC and TCGA breast cancer cohorts. Tukey-type boxplots show median and interquartile level values, and the ANOVA test was used to calculate *p* values. (b) Tumors with low (blue) and high (red) inflammatory scores in both cohorts of patients with each subgroup are compared for survival DSS, DFS, and OS of inflammatory score high and low inflammatory groups in each breast cancer subtype of METABRIC cohort. Log-rank test used to calculate *p* values to compare two groups with Kaplan-Meier survival curves.

In the whole breast cancer cohort, inflammation was associated with aggressive tumor and worse outcome most likely because inflammatory score high group included most of TNBC, which is known to be the most aggressive subtype. Therefore, it was of interest to study the association of inflammation and tumor aggressiveness within each subtype. We used Nottingham pathological grade as a surrogate of tumor aggressiveness and found that there was no association between grade and inflammatory score in any of subtype (Figure 2(a)). We also investigated the association of the inflammatory score with age in TNBC subtype, but we found no significant association in neither TCGA nor METABRIC cohorts (Figure S2). Interestingly, high inflammatory score was significantly associated with better survival in TNBC (DSS, DFS, OS; *p* = 0.014, 0.062, 0.015, respectively), but not in the other subtypes (none were statistically significant) (Figure 2(b)). These findings suggest that extremely high inflammation in TNBC was associated with favorable cancer biology.

### 3.3. High Inflammatory Pathway Score Significantly Enriched Both Favorable and Unfavorable Immune-Related Gene Sets in TNBC

**Figure 3:**
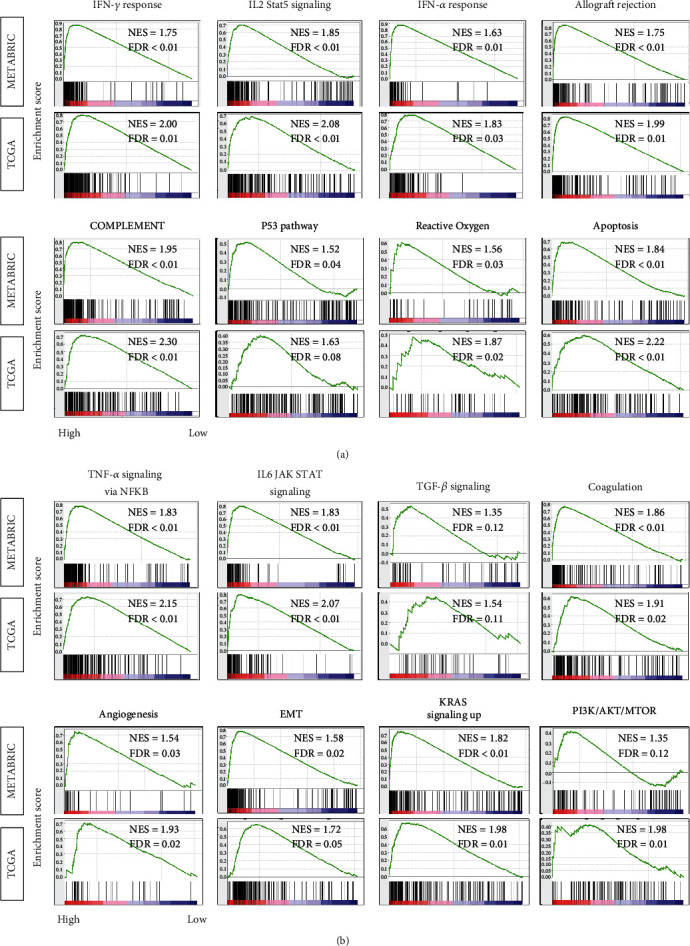
Gene Set Enrichment Assay (GSEA) of high inflammatory pathway score in triple-negative breast cancer. Enrichment plots are shown for (a) favorable immune-related and (b) unfavorable Hallmark gene sets for which highly enriched in the high inflammatory pathway score compared to low-score group in both the TCGA and METABRIC cohorts, along with normalized enrichment score (NES) and false discovery rate (FDR). The statistical significance of GSEA was determined using FDR of 0.25.

We hypothesized that TNBC with a high inflammatory score is associated with favorable antitumor immune activity, since it was associated with better survival. To test this hypothesis, gene set enrichment analysis (GSEA) of the Hallmark gene sets was performed in TNBC. Tumors with a high inflammatory pathway score significantly enriched many favorable immune-related Hallmark gene sets, such as *IFN*-*γ* response, *IL2 stat5* signaling, *IFN*-*α* response, Allograft rejection, Complement, p53 pathway, Reactive Oxygen, and Apoptosis (Figure 3(a)). Interestingly, high inflammatory score enriched unfavorable pathway gene sets as well, such as *TNF*-*α* signaling via *NFkB*, *IL6 JAK STAT* signaling, *TGF*-*β* signaling, Coagulation, Angiogenesis, Epithelial-mesenchymal transition (EMT), *KRAS* signaling up, and *PI3K AKT MTOR* signaling (Figure 3(b)). These findings were remarkably consistent in both METABRIC and TCGA cohorts. These results suggest that inflammation is associated with both favorable and unfavorable reactions which are most likely intricately intertwined and led to context-dependent clinical outcome in TNBC.

### 3.4. High Inflammatory Pathway Score Is Associated with Infiltration of Favorable Anticancerous Immune Cells in TNBC

**Figure 4:**
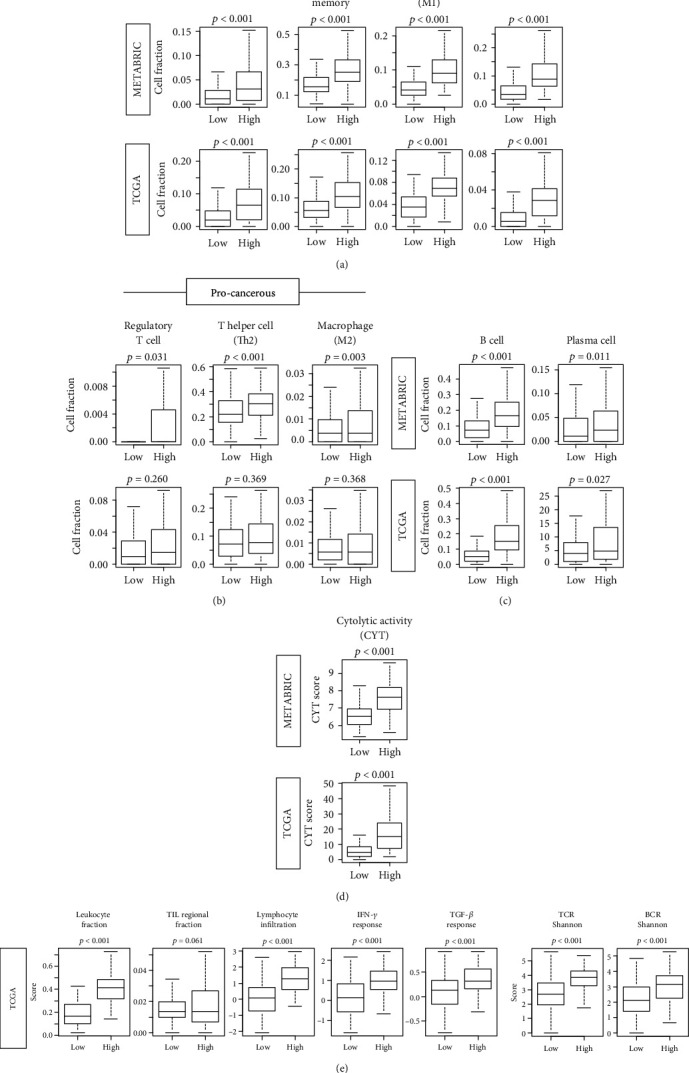
Composition of tumor infiltrating immune cells and immune function scores by high or low inflammatory scores in METABRIC and TCGA cohorts. (a) anticancerous immune cells including CD8 T cells, CD4 memory T cells, M1 macrophages, and dendritic cells (DC); (b) procancerous immune cells including regulatory T cells, Type 2 helper T cells (Th2), and M2 macrophages; and (c) B cells and plasma cells in the TCGA and METABRIC cohorts. Comparison of high vs. low inflammatory scores in (d) cytolytic activity score (CYT). (e) Leukocyte fraction, tumor infiltrating lymphocyte (TIL) regional fraction, lymphocyte infiltration, Interferon-gamma (IFN-*γ*) response, T cell receptor (TCR) Shannon, TCR Richness, B cell receptor (BCR) Shannon, *BCR* Richness, and transforming growth factor-beta (*TGF*-*β*) response. Inflammation score in each cohort were divided into low and high groups by the median value. Tukey-type boxplots show median and interquartile level values, and the ANOVA test is used to calculate *p* values.

Since high inflammatory score group have enriched immune response gene sets, it was of interest to determine which types of immune cells are infiltrating in inflammation high TNBC tumors. We showed the interplay of cancer cell with anti- and procancer immune cells in supplementary Figure S3. xCell algorithm, which estimates immune cell composition by gene expression data of a bulk tumor, was utilized. We found that significantly higher fractions of anticancerous immune cells: CD8^+^ T cell, CD4^+^ memory T cell, M1 macrophage, and dendritic cell (DC), as well as B cell and plasma cell, were infiltrated in the high inflammatory pathway score group in both METABRIC and the TCGA cohorts (Figures 4(a) and 4(c)). Although high inflammatory score group was also associated with procancerous immune cells, Regulatory T cell, T helper type 2 (Th2) cell, and M2 macrophage, in METABRIC cohort, this result was not reproduced in TCGA cohort (Figure 4(b)).

To further investigate the relationship between the inflammatory pathway score and cancer immunity, we further analyzed its association with several other scores that have been previously reported [50, 51]. Cytolytic activity score (CYT) was significantly enhanced in inflammatory score high group consistently in both METABRIC and TCGA, which suggest that there is overall immune cell killing in inflammation high group (Figure 4(c)). Leukocyte fraction, tumor infiltrating lymphocyte (TIL) regional fraction, Lymphocyte infiltration, and *IFN-γ* response as well as *TGF-β* response score were all significantly higher in high inflammatory score group, consistent with GSEA results in Figures 3 and 4(d). Furthermore, high inflammatory score was significantly associated with high T cell receptor (TCR) and B cell receptor (BCR) Shannon score, which represent the TCR and BCR diversity that is thought to be beneficial (Figure 4(d)). Taken together, high inflammatory pathway score TNBC was associated with enhanced immune response and favorable anticancerous immune cells compared to the low score group.

### 3.5. The Inflammatory Pathway Score Was Not Associated with Neoadjuvant Chemotherapy (NAC) Response, Whereas It Was with Immune Checkpoint Molecule Expressions

**Figure 5:**
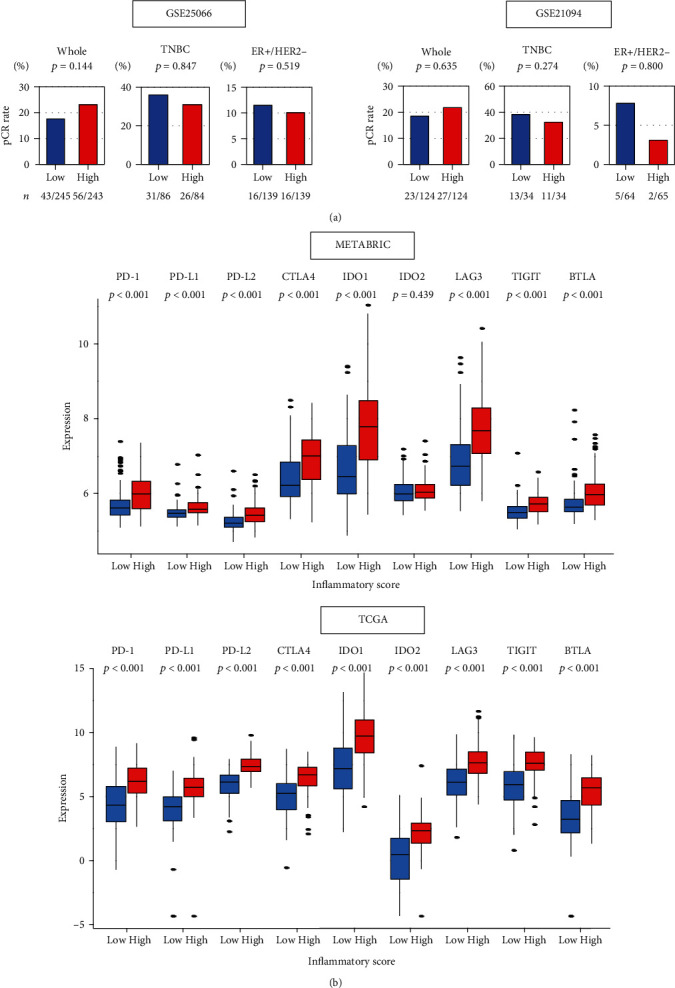
Relationships between the inflammatory score and neoadjuvant chemotherapy or expression of immune checkpoint molecules. (a) Percentage of achievement of pathological complete response (pCR) between low (blue bar) and high (red bar) inflammatory score in whole, TNBC and ER+/HER2- in the GSE25066 (*n* = 467) and GSE20194 (*n* = 248) breast cancer cohorts that underwent neoadjuvant chemotherapy. The number of patients who achieved pCR is shown below the plots. Fisher's exact test is used to compare pCR rates between two groups. (b) Comparison of low (blue) and high (red) inflammatory score groups in gene expression of immune checkpoint molecules (log_2_ transcripts per million) in METABRIC and TCGA cohorts. Inflammatory score in each cohort was divided into low and high groups by the median values. Tukey-type boxplots show median and interquartile level values, and the ANOVA test is used to calculate *p* values. *BTLA*: B- and T-lymphocyte attenuator; *CTLA4*: cytotoxic T-lymphocyte-associated protein 4; *IDO1/2*: indoleamine dioxygenase 1/2; *LAG3*: lymphocyte activation gene 3; *PD-1*: programmed death-1; *PD-L1*: programmed death ligand 1; *TIGIT*: tyrosine-based inhibitory motif domain.

It is well known that tumors with high infiltration of TILs have a better response to NAC [52–54]. Given the results of Figures 3 and 4, we hypothesized that the inflammatory pathway score would be a predictive biomarker for NAC response. However, contrary to our expectations, the score did not associate with the pathological complete response (pCR) rate in NAC in neither the whole breast cancer cohort nor in any subtype (Figure 5(a)).

Lately, there is a growing interest in use of immune checkpoint inhibitors for breast cancer [55]. Thus, the relationship between the score and immune checkpoint molecule expressions was examined in METABRIC and TCGA cohorts. Expression of most immune checkpoint molecules that are targeted by specific inhibitors, such as *PD-*1, *PD-L1*, and *CTLA-4*, was positively associated with the inflammatory score in TNBC in both cohorts (Figure 5(b)). These results suggest that the inflammatory pathway score high tumors may associate with response to immune checkpoint inhibitors, whereas the score did not with NAC response.

## 4. Discussion

Here, we examined the association between the level of inflammation, determined as GSVA score of the Hallmark inflammatory response gene set, with cancer aggressiveness, survival, and treatment response in breast cancer using bulk tumor transcriptomes from multiple cohorts of breast cancer patients. In whole breast cancer cohort, the tumors with a high inflammatory pathway score were associated with aggressive clinical characteristics, such as worse DSS, higher Nottingham histological grade, and younger age at diagnosis. Inflammatory score was significantly high in TNBC as well as basal and normal subtypes compared with the other subtypes, which suggest that the detrimental effect of high level of inflammation may be because it includes an aggressive subtype. On the contrary, high inflammatory score within TNBC was significantly associated with better DSS and OS. TNBC with high inflammatory pathway score enriched not only anticancerous immune pathway such as IFN-*α*, IFN-*γ* response, IL-2/STAT5 signaling, Allograft rejection, Complement, p53 pathway, Reactive Oxygen, and Apoptosis but also procancerous pathways such as TNF-*α* signaling via NFkB, IL6 JAK STAT signaling, TGF-*β* signaling, Coagulation, Angiogenesis, EMT, *KRAS* signaling up, and PI3K AKT MTOR signaling. A high inflammatory pathway score was associated with mainly favorable anticancerous immune cell infiltration in the tumor immune microenvironment. Furthermore, high inflammatory pathway score TNBC were also associated with various other immune-related scores, including leukocyte fraction, TIL regional fraction, Lymphocyte infiltration, IFN-*γ* response, and TGF-*β* response scores as well as CYT. Although the inflammatory pathway score was not associated with neoadjuvant treatment response, it was associated with expressions of immune checkpoint molecules.

Whether inflammation aggravates or conciliates breast cancer is not generalizable due to the complexity of the mechanism. Our group has previously reported that a lipid mediator, S1P, links inflammation and cancer due to the amplification loop of IL6 and NFkB [3, 6]. This led to our following discovery that S1P plays a critical role in inflammation-mediated breast cancer progression and metastasis [5]. In the current study, we demonstrated for the first time in human breast cancer that elevated inflammation was significantly associated with high expression of S1P-signaling genes, such as *SphK1* and *S1PR1*.

In the current study, we have shown that the clinical impact of inflammation is context dependent in breast cancer, and subtype-specific evaluation is necessary. We found that clinical relevance of inflammation was opposite between the whole breast cancer cohort and TNBC. Three major subtypes of breast cancer (estrogen receptor (ER) and progesterone receptor- (PR) positive, human epidermal growth factor receptor 2 (HER2) overexpression, and TNBC) have different clinical characteristics and outcomes [56]. This may be due to the differences in infiltration of immune cells that impact survival and treatment response [57]. Specifically, TNBC, the most aggressive subtype, is well known to have elevated inflammation and immune cell infiltration to the point that it was described by some as an “immunomodulatory subtype” [58, 59]. We found that elevated inflammation was associated with aggressive cancer biology and poor survival in whole breast cancer cohort most likely because it reflected inclusion of TNBC. However, elevated inflammation among TNBC was associated with better survival most likely due to enrichment of immune reaction pathway due to infiltration of favorable anticancerous immune cells such as high IFN-*γ* response and cytolytic activity and infiltration of CD8^+^ T cells. Our results suggest that inflammation can evoke either favorable or unfavorable immune reactions, and its clinical impact depends on which immune reaction occurs in which tumor, although our results indicate that extreme inflammation may be beneficial in breast cancer. It further indicates that it is important to analyze the inflammatory status in each cancer patient, rather than simply assessing the presence or absence of inflammation for the treatment of breast cancer. Our inflammatory pathway score that measures the amount of inflammation of each patient tumor may become a useful tool for future breast cancer management.

Immune checkpoint inhibitors such as anti-*PD-1/PD-L1* and *CTLA-4* antibodies have added another therapeutic approach, immunotherapy, to fight breast cancer [55]. Following the Impassion130 trial, the US Food and Drug Administration approved Atezolizumab, an anti-*PD-L1* antibody, in conjunction with nab-paclitaxel for metastatic TNBC [60, 61]. However, only a small portion of breast cancer patients respond to immune checkpoint inhibitors [62]. Therefore, the appropriate patient selection to treat only the patients who are likely to respond is the key for most efficient management strategy not only to maximize the benefit of immunotherapy but also to avoid side effects and unnecessary medical expense. In our study, we found that the high inflammatory pathway score was significantly associated with the expression of major immune checkpoint molecules; thus, we cannot help but speculate that inflammatory pathway score may be useful in predicting response to immune checkpoint inhibitors.

This is the first study to demonstrate that inflammation is associated with worse outcome in whole breast cancer, but with better outcome in TNBC using the TCGA, METABRIC, and GEO cohorts. Although utilization of the large number of publicly available independent human breast cancer gene expression data sets allows consistent and reproducible results, limitation of our study is that it is a retrospective analysis. Multiple datasets from different sources have validated our findings, but additional experiments are needed to prove the mechanism. Our inflammatory score needs to be further verified in future prospective clinical trials and molecular biology researches to be used in clinical practice. And further analysis is needed to study the relationship between obesity and inflammation using decent sample size.

## 5. Conclusion

Inflammation was associated with worse outcome in the whole breast cancer cohort, but with better outcome in TNBC, which was associated with favorable anticancerous immune response and immune cell infiltrations.

## Data Availability

All data relevant to the study are included in the article.

## Abbreviations

AJCC: American Joint Committee on Cancer
DFS: Disease-free survival
DSS: Disease-specific survival
ER: Estrogen receptor
FDR: False discovery rate
GSVA: Gene set variation analysis
HER2: Human epidermal growth factor receptor 2
METABRIC: Molecular Taxonomy of Breast Cancer International Consortium
NES: Normalized enrichment score
OS: Overall survival
pCR: Pathological complete response
PFS: Progression-free survival
TCGA: The Cancer Genome Atlas
TNBC: Triple-negative breast cancer.

## Supplementary Materials

Supplementary Materials Table S1: member genes of the Hallmark inflammatory response gene set. Figure S1: association of the inflammation score with American Joint Committee on Cancer (AJCC) T- and N- category, and body mass index (BMI) in the TCGA cohort. Figure S2: association of the inflammation score with age in TNBC patients. Figure S3: scheme of the interplay between cancer cells and anti-/procancer immune cells.
