# Supplementary material for: Inflammation Is Associated with Worse Outcome in the Whole Cohort but with Better Outcome in Triple-Negative Subtype of Breast Cancer Patients
Source: J Immunol Res. 2020 Dec 8;2020:5618786. doi: 10.1155/2020/5618786 (PMC7787871; doi:10.1155/2020/5618786)
Supplement: Supplementary Materials — Table S1: member genes of the Hallmark inflammatory response gene set. Figure S1: association of the inflammation score with American Joint Committee on Cancer (AJCC) T- and N- category, and body mass index (BMI) in the TCGA cohort. Figure S2: association of the inflammation score with age in TNBC patients. Figure S3: scheme of the interplay between cancer cells and anti-/procancer immune cells. [file 5618786.f1.docx]

SUPPLEMENTAL MATERIALS

Oshi et al. “Inflammation is associated with worse outcome in the whole cohort but with better outcome in triple negative subtype of breast cancer patients”

Content

Table S1: Member genes of the Hallmark inflammatory response gene set.

Figure S1: Association of the inflammation score with American Joint Committee on Cancer (AJCC) T- and N- category, and body mass index (BMI) in the TCGA cohort.

Figure S2: Association of the inflammation score with age in TNBC patients.

Figure S3: Scheme of the interplay between cancer cells and anti-/pro-cancer immune cells.

| *Table S1: Member genes of the Hallmark inflammatory response gene set.*  *HALLMARK_INFLAMMATORY_RESPONSE* | | | |
| --- | --- | --- | --- |
|  | |  |  |
| ABCA1 | ATP Binding Cassette subfamily A Member 1 |  |  |
| ABI1 | ABI Interactor 1 |  |  |
| ACVR1B | Activin Receptor Type 1B |  |  |
| ACVR2A | Activin Receptor Type 2A |  |  |
| ADGRE1 | Adhesion G Protein Coupled Receptor E1 |  |  |
| ADM | Adrenomedullin |  |  |
| ADORA2B | Adenosine A2B Receptor |  |  |
| ADRM1 | Adhesion Regulating Molecule 1 |  |  |
| AHR | Aryl Hydrocarbon Receptor |  |  |
| APLNR | Apelin Receptor |  |  |
| AQP9 | Aquaporin-9 |  |  |
| ATP2A2 | ATPase Sarcoplasmic/Endoplasmic Reticulum Ca2+ Transporting 2 |  |  |
| ATP2B1 | ATPase Plasma Membrane Ca2+ Transporting 1 |  |  |
| ATP2C1 | ATPase Secretory Pathway Ca2+ Transporting 1 |  |  |
| AXL | AXL Receptor Tyrosine kinase |  |  |
| BDKRB1 | Bradykinin Receptor B1 |  |  |
| BEST1 | Bestrophin-1 |  |  |
| BST2 | Bone Marrow stromal Cell Antigen 2 |  |  |
| BTG2 | BTG Anti-Proliferation Factor 2 |  |  |
| C3AR1 | Complement C3a Receptor 1 |  |  |
| C5AR1 | Complement C5a Receptor 1 |  |  |
| CALCRL | Calcitonin Receptor Like Receptor |  |  |
| CCL17 | C-C motif Chemokine Ligand 17 |  |  |
| CCL2 | C-C Motif Chemokine Ligand 2 |  |  |
| CCL20 | C-C Motif Chemokine Ligand 20 |  |  |
| CCL22 | C-C Motif Chemokine Ligand 22 |  |  |
| CCL24 | C-C Motif Chemokine Ligand 24 |  |  |
| CCL5 | C-C Motif Chemokine Ligand 5 |  |  |
| CCL7 | C-C Motif Chemokine Ligand 7 |  |  |
| CCR7 | C-C Motif Chemokine Receptor 7 |  |  |
| CCRL2 | C-C Motif Chemokine Receptor Like 2 |  |  |
| CD14 | Cluster of Differentiation 14 |  |  |
| CD40 | Cluster of Differentiation 40 |  |  |
| CD48 | Cluster of Differentiation 48 |  |  |
| CD55 | Cluster of Differentiation 55 |  |  |
| CD69 | Cluster of Differentiation 69 |  |  |
| CD70 | Cluster of Differentiation 70 |  |  |
| CD82 | Cluster of Differentiation 82 |  |  |
| CDKN1A | Cyclin Dependent Kinase Inhibitor 1A |  |  |
| CHST2 | Carbohydrate Sulfotransferase 2 |  |  |
| CLEC5A | C-type Lectin Domain Family 5 Member A |  |  |
| CMKLR1 | Chemokine Like Receptor 1 |  |  |
| CSF1 | Colony Stimulating Factor 1 |  |  |
| CSF3 | Colony Stimulating Factor 3 |  |  |
| CSF3R | Colony Stimulating Factor 3 Receptor |  |  |
| CX3CL1 | C-X3-C Motif Chemokine Ligand 1 |  |  |
| CXCL10 | C-X-C Motif Chemokine ligand 10 |  |  |
| CXCL11 | C-X-C Motif Chemokine ligand 11 |  |  |
| CXCL6 | C-X-C Motif Chemokine ligand 6 |  |  |
| CXCL8 | C-X-C Motif Chemokine ligand 8 |  |  |
| CXCL9 | C-X-C Motif Chemokine ligand 9 |  |  |
| CXCR6 | C-X-C Chemokine Receptor Type 6 |  |  |
| CYBB | Cytochrome B-245 Beta Chain |  |  |
| DCBLD2 | Discoidin, CUB and LCCL Domain Containing 2 |  |  |
| EBI3 | Epstein-Barr Virus Induced 3 |  |  |
| EDN1 | Endothelin 1 |  |  |
| EIF2AK2 | Eukaryotic Translation Initiation Factor 2 Alpha Kinase 2 |  |  |
| EMP3 | Epithelial membrane Protein 3 |  |  |
| EREG | Epiregulin |  |  |
| F3 | Coagulation Factor III |  |  |
| FFAR2 | Free fatty Acid Receptor 2 |  |  |
| FPR1 | Formyl Peptide Receptor 1 |  |  |
| FZD5 | Frizzled 5 |  |  |
| GABBR1 | Gamma-Aminobutyric Acid Type B Receptor Subunit 1 |  |  |
| GCH1 | GTP Cyclohydrolase 1 |  |  |
| GNA15 | Guanine Nucleotide Binding Protein Subunit Alpha 15 |  |  |
| GNAI3 | Guanine Nucleotide Binding Protein Subunit Alpha I3 |  |  |
| GP1BA | Glycoprotein lb Platelet Subunit Alpha |  |  |
| GPC3 | Glypican 3 |  |  |
| GPR132 | G Protein Coupled Receptor 132 |  |  |
| GPR183 | G Protein Coupled Receptor 183 |  |  |
| HAS2 | Hyaluronan Synthase 2 |  |  |
| HBEGF | Heparin Binding EGF Like Growth Factor |  |  |
| HIF1A | Hypoxia Inducible Factor 1 Alpha |  |  |
| HPN | Hepsin |  |  |
| HRH1 | Histamine H1 |  |  |
| ICAM1 | Intercellular Adhesion Molecule 1 |  |  |
| ICAM4 | Intercellular Adhesion Molecule 4 |  |  |
| ICOSLG | Inducible T Cell Costimulator Ligand |  |  |
| IFITM1 | Interferon Induced Transmembrane Protein 1 |  |  |
| IFNAR1 | Interferon Alpha and Beta Receptor Subunit 1 |  |  |
| IFNGR2 | Interferon Gamma Receptor 2 |  |  |
| IL10 | Interleukin 10 |  |  |
| IL10RA | Interleukin 10 Receptor Subunit Alpha |  |  |
| IL12B | Interleukin 12 Subunit Beta |  |  |
| IL15 | Interleukin 15 |  |  |
| IL15RA | Interleukin 15 Receptor Subunit Alpha |  |  |
| IL18 | Interleukin 18 |  |  |
| IL18R1 | Interleukin 18 Receptor 1 |  |  |
| IL18RAP | Interleukin 18 Receptor Accessory Protein |  |  |
| IL1A | Interleukin 1 Alpha |  |  |
| IL1B | Interleukin 1 Beta |  |  |
| IL1R1 | Interleukin 1 Receptor 1 |  |  |
| IL2RB | Interleukin 2 Receptor Subunit Beta |  |  |
| IL4R | Interleukin 4 Receptor |  |  |
| IL6 | Interleukin 6 |  |  |
| IL7R | Interleukin 7 Receptor |  |  |
| INHBA | Inhibin Subunit Beta A |  |  |
| IRAK2 | Interleukin 1 Receptor Associated Kinase 2 |  |  |
| IRF1 | Interferon Regulatory Factor 1 |  |  |
| IRF7 | Interferon Regulatory Factor 7 |  |  |
| ITGA5 | Integrin Alpha 5 |  |  |
| ITGB3 | Integrin Subunit Beta 3 |  |  |
| ITGB8 | Integrin Subunit Beta 8 |  |  |
| KCNA3 | Potassium Voltage Gated Channel Subfamily A Member 3 |  |  |
| KCNJ2 | Potassium Inwardly Rectifying Channel Subfamily J Member 2 |  |  |
| KCNMB2 | Potasium Calcium Activated Channel Subfamily M Regulatory Beta Subunit 2 |  |  |
| KIF1B | Kinesin Family Member 1B |  |  |
| KLF6 | Krueppel Like Factor 6 |  |  |
| LAMP3 | Lysosome Associated Membrane Glycoprotein 3 |  |  |
| LCK | Lymphocyte Specific Protein Tyrosine Kinase |  |  |
| LCP2 | Lymphocyte Cytosolic Protein 2 |  |  |
| LDLR | Low Density Lipoprotein Receptor |  |  |
| LIF | Leukemia Inhibitory Factor |  |  |
| LPAR1 | Lysophosphatidic Acid Receptor 1 |  |  |
| LTA | Lymphotoxin Alpha |  |  |
| LY6E | Lymphocyte Antigen 6E |  |  |
| LYN | LYN Proto Oncogene |  |  |
| MARCO | Macrophage Receptor |  |  |
| MEFV | Mediterranean Fever |  |  |
| MEP1A | Meprin A Subunit Alpha |  |  |
| MET | MesenchymalEpithelial Transition Proto Oncogene |  |  |
| MMP14 | Matrix Metalloproteinase 14 |  |  |
| MSR1 | Macrophage Scavenger Receptor 1 |  |  |
| MXD1 | MAX Dimerization Protein 1 |  |  |
| MYC | MYC Proto Oncogene |  |  |
| NAMPT | Nicotinamide Phosphoribosyltransferase |  |  |
| NDP | Norrie Disease Protein |  |  |
| NFKB1 | Nuclear factor Kappa B Subunit 1 |  |  |
| NFKBIA | NFkB Inhibitor Alpha |  |  |
| NLRP3 | NLR Family Pyrin Domain Containing 3 |  |  |
| NMI | N Myc and STAT Interactor |  |  |
| NMUR1 | Nueuromedin U Receptor 1 |  |  |
| NOD2 | Nucleotide Binding oligomerization Domain Containing Protein 2 |  |  |
| NPFFR2 | Neuropeptide FF Receptor 2 |  |  |
| OLR1 | Oxidized Low Density lipoprotein Receptor 1 |  |  |
| OPRK1 | Opioid Receptor Kappa 1 |  |  |
| OSM | Oncostatin M |  |  |
| OSMR | Oncostatin M Receptor |  |  |
| P2RX4 | P2X Purinoceptor 4 |  |  |
| P2RX7 | P2X Purinoceptor 7 |  |  |
| P2RY2 | P2Y Purinoceptor 2 |  |  |
| PCDH7 | Protocadherin 7 |  |  |
| PDE4B | Phosphodiesterase 4B |  |  |
| PDPN | Podoplanin |  |  |
| PIK3R5 | Phosphoinositide 3 Kinase Regulatory Subunit 5 |  |  |
| PLAUR | Plasminogen Activator, Urokinase Receptor |  |  |
| PROK2 | Prokineticin 2 |  |  |
| PSEN1 | Presenilin 1 |  |  |
| PTAFR | Platelet Activating Factor Receptor |  |  |
| PTGER2 | Prostaglandin E Receptor 2 |  |  |
| PTGER4 | Prostaglandin E Receptor 4 |  |  |
| PTGIR | Prostaglandin I2 |  |  |
| PTPRE | Protein Tyrosine Phosphatase Receptor Type E |  |  |
| PVR | PVR Cell Adhesion Molecule |  |  |
| RAF1 | Raf 1 Proto Oncogene |  |  |
| RASGRP1 | RAS Guanyl Releasing Protein 1 |  |  |
| RELA | V-rel Avian Reticuloendotheliosis Viral Oncogene Homolog A |  |  |
| RGS1 | Regulator of G Protein Signaling 1 |  |  |
| RGS16 | Regulator of G Protein Signaling 16 |  |  |
| RHOG | Ras Homology Growth Related |  |  |
| RIPK2 | Receptor interacting Serine and Threonin Protein Kinase 2 |  |  |
| RNF144B | Ring Finger Protein 144B |  |  |
| ROS1 | ROS Proto Oncogene 1 |  |  |
| RTP4 | Receptor Transporter Protein 4 |  |  |
| SCARF1 | Scavenger Receptor Class F Member 2 |  |  |
| SCN1B | Sodium Channel Subunit Beta 1 |  |  |
| SELE | Selection E |  |  |
| SELENOS | Selenoprotein S |  |  |
| SELL | Selectin L |  |  |
| SEMA4D | Semaphorin 4D |  |  |
| SERPINE1 | Serpin Family E Member 1 |  |  |
| SGMS2 | Sphingomyelin Synthase 2 |  |  |
| SLAMF1 | Signaling Lymphocytic Activation Molecule 1 |  |  |
| SLC11A2 | Solute Carrier Family 11 Member 2 |  |  |
| SLC1A2 | Solute Carrier Family 1 Member 2 |  |  |
| SLC28A2 | Solute Carrier Family 28 Member 2 |  |  |
| SLC31A1 | Solute Carrier Family 31 Member 1 |  |  |
| SLC31A2 | Solute Carrier Family 31 Member 2 |  |  |
| SLC4A4 | Solute Carrier Family 4 Member 4 |  |  |
| SLC7A1 | Solute Carrier Family 7 Member 1 |  |  |
| SLC7A2 | Solute Carrier Family 7 Member 2 |  |  |
| SPHK1 | Sphingosine Kinase 1 |  |  |
| SRI | Sorcin |  |  |
| STAB1 | Stabilin 1 |  |  |
| TACR1 | Tachykinin Receptor 1 |  |  |
| TACR3 | Tachykinin Receptor 3 |  |  |
| TAPBP | TAP Binding Protein |  |  |
| TIMP1 | TIMP Metallopeptidase Inhibitor 1 |  |  |
| TLR1 | Toll Like Receptor 1 |  |  |
| TLR2 | Toll Like Receptor 2 |  |  |
| TLR3 | Toll Like Receptor 3 |  |  |
| TNFAIP6 | Tumor Necrosis Factor Alpha Induced Protein 6 |  |  |
| TNFRSF1B | Tumor Necrosis Factor Receptor Superfamily Member 1B |  |  |
| TNFRSF9 | Tumor Necrosis Factor Receptor Superfamily Member 9 |  |  |
| TNFSF10 | Tumor Necrosis Factor Superfamily Member 10 |  |  |
| TNFSF15 | Tumor Necrosis Factor Superfamily Member 15 |  |  |
| TNFSF9 | Tumor Necrosis Factor Superfamily Member 9 |  |  |
| TPBG | Trophoblast Glycoprotein |  |  |
| VIP | Vasoactive Intestinal Peptide |  |  |


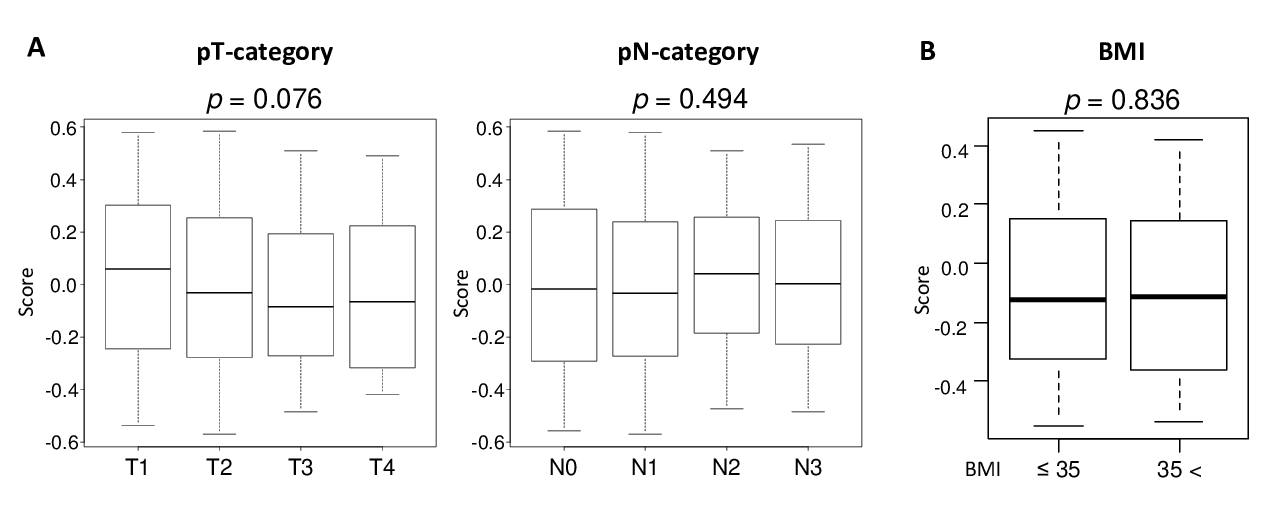


**Figure S1:** Association of the inflammation score with American Joint Committee on Cancer (AJCC) T- and N- category, and body mass index (BMI) in the TCGA cohort. Boxplots of the inflammation score by pathological AJCC T- and N- category, and (B) BMI low (*n* = 54) and high (*n* = 27) group in the TCGA cohort.

**
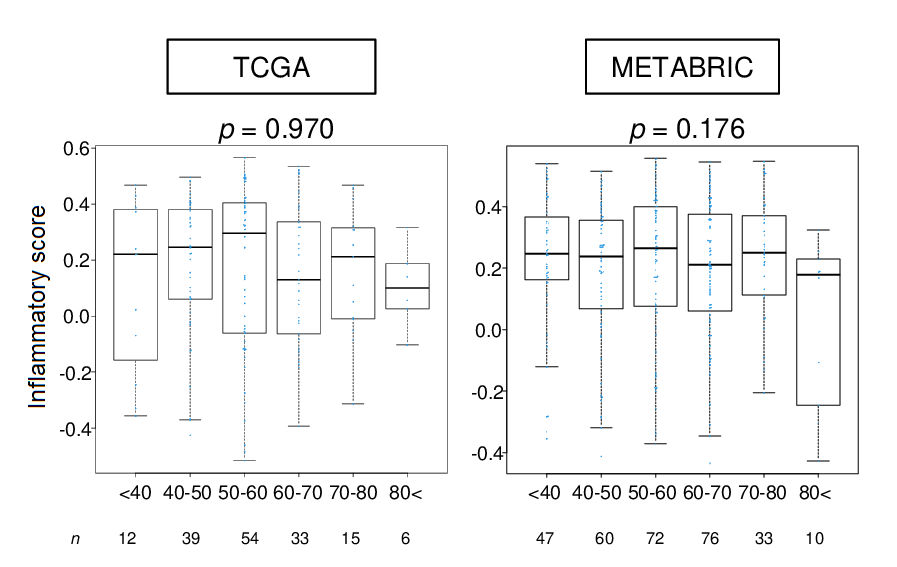
**

**Figure S2:** Association of the inflammation score with age in TNBC patients. Boxplots of the inflammation score by age at diagnosis (< 40yo, 40-50yo, 50-60 yo, 60-70 yo, 70-80 yo, 80 yo <) in the TCGA and METABRIC cohort.

**
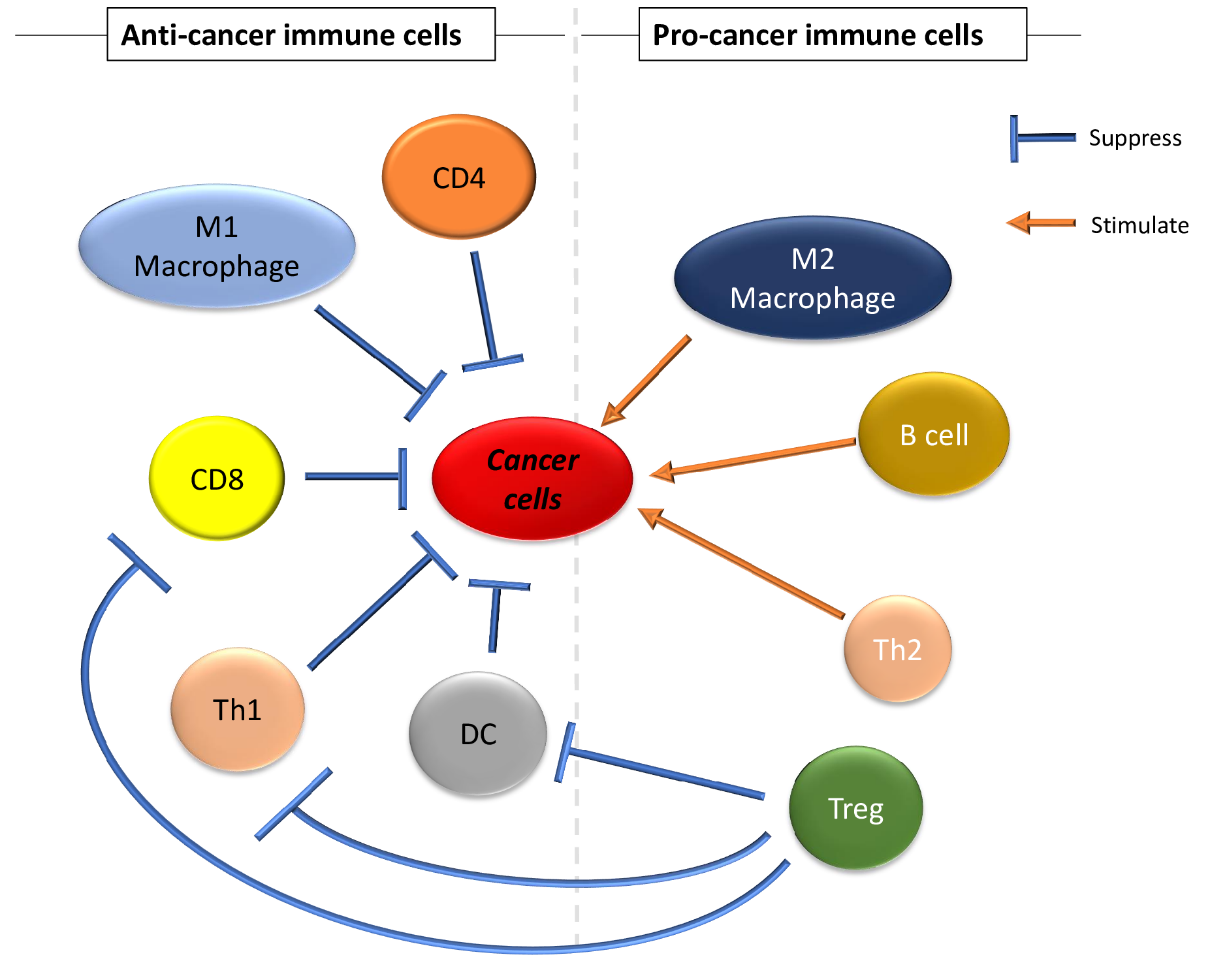
**

**Figure S3:** Scheme of the interplay between cancer cells and anti-/pro-cancer immune cells.
